# The impact of video speed on the decision-making process of sports officials

**DOI:** 10.1186/s41235-018-0105-8

**Published:** 2018-06-11

**Authors:** Jochim Spitz, Pieter Moors, Johan Wagemans, Werner F. Helsen

**Affiliations:** 1 Department of Movement Sciences, Laboratory of Perception and Performance, Movement Control and Neuroplasticity Research Group, University of Leuven (KU Leuven), Tervuursevest 101, (box 1501), B – 3001, Leuven, Belgium; 2 Department of Brain & Cognition, Laboratory of Experimental Psychology, University of Leuven (KU Leuven), Tiensestraat 102, (box 3711), B – 3000, Leuven, Belgium

**Keywords:** Decision-making, Visual perception, Motion perception, Slow motion, Association football

## Abstract

There is an increasing trend in association football (soccer) to assist referees in their decision-making with video technology. For decisions such as whether a goal has been scored or which player actually committed a foul, video technology can provide more objective information and be valuable to increase decisional accuracy. It is unclear, however, to what extent video replays can aid referee decisions in the case of foul-play situations in which the decision is typically more ambiguous. In this study, we specifically evaluated the impact of slow-motion replays on decision-making by referees. To this end, elite referees of five different countries (n = 88) evaluated 60 different foul-play situations taken from international matches, replayed in either real time or slow motion. Our results revealed that referees penalized situations more severely in slow motion compared to real time (e.g. red card with a yellow card reference decision). Our results provide initial evidence that video replay speed can have an important impact on the disciplinary decision given by the referee in case of foul play. The study also provides a real-life test-case for theories and insights regarding causality perception.

## Significance

In sporting events, high-speed cameras are frequently used to provide slow-motion replays of important game situations in every detail. Fans and sport commentators have immediate access to this footage and they increasingly rely on it to scrutinize referee decisions. Furthermore, the International Football Association Board agreed to introduce experiments with video assistant referees who can rely on video clips in slow motion and/or real time to evaluate and review referee decisions on the spot. Although referees on the field of play have to decide in real time, these slow-motion replays are frequently adopted as an objective representation and comparison standard. The current study demonstrates that there is a biasing potential of slow-motion replays when assessing foul-play situations. Slow motion can, for example, increase the probability for penalizing an offender with a red card instead of a yellow card. These findings are of particular relevance for all stakeholders in sports and refereeing. Slow motion is a separate viewing mode and it can alter the result of crucial decisions, thereby impacting on the final outcome or perceived justice throughout and after a game. Consequently, we discuss clear and consistent guidelines regarding when and how slow-motion replays can be used and the link with basic perceptual and cognitive functions.

## Background

Referees in team sports are responsible for interpreting and enforcing the specific rules of the sport from a neutral point of view. They have to take into account several sources of information and make up their mind to maintain fair play and protect the players’ safety (Bar-Eli, Plessner, & Raab, 2011; MacMahon et al., 2014). In association football (soccer), a referee decision might have an impact on the outcome of the game, for instance when a player is sent off or a goal is denied. The performance of referees can thus have far-reaching consequences for players, clubs, fans, and other stakeholders. Given these responsibilities, a correct, adequate, consistent, and uniform implementation of the Laws of the Game of the Fédération Internationale de Football Association (FIFA, 2016) is crucial.

With the continued spread of high-definition cameras at better positions together with fans and sport commentators having immediate access to this footage, it is increasingly likely that a referee decision will be recorded and scrutinized in depth in post-match analyses and even during the game. For unambiguous situations, video technology can provide more objective and accurate information. For instance, goal-line technology enables the possibility to accurately determine whether a ball crossed the goal line or not. In other, typically more ambiguous situations (e.g. foul-play situations), judgments of referees require subjective evaluations to determine the intentionality of the offence and the disciplinary sanction for the offending player. According to the Laws of the Game (FIFA, 2016), several factors have to be considered, such as the element of intent, the speed of the player’s action, and the safety of the opponent. Helsen and Bultynck (2004) found that approximately one-third of the total decisions made by association football referees are related to foul situations.

Fans, players, sport commentators, and journalists increasingly rely on video replays, which are often played in slow motion, to evaluate and discuss the referee decision. During decision-training and feedback sessions, referees are also exposed to video replays in slow motion. The use of video technology has already found its way into several sports such as field and ice hockey, cricket, tennis, and basketball to assist the referee and improve decisional accuracy. Here, video technology is used to evaluate whether a goal was validly scored, to determine whether a ball was in or out, or to reconsider foul-play situations. Interestingly, most decisions for which video technology is currently used involve situations for which fairly to clearly objective criteria exist. As such, video technology can effectively increase the accuracy of referee decisions during the game by allowing certain situations to be reviewed from various perspectives and/or replay speeds. Indeed, in field hockey and cricket, video referees have access to replays at different replay speeds and they can use (a combination of) different speeds to arrive at a decision.

Although slow motion is commonly used in video refereeing, it is currently unclear to what extent the use of variable video speeds impacts decision-making by referees. The relevance of this question was emphasized by a recent study of Caruso, Burns, and Converse (2016). Here, the authors examined the impact of slow-motion video evidence on judgment of responsibility for harmful actions in the courtroom. Participants watched surveillance footage of an attempted robbery with the store clerk being shot by an assailant. Video footage was available in real time and slow motion and it was concluded that slow motion systematically increased perceptions of premeditation. That is, an action was perceived as more intentional and the odds of unanimous first-degree murder verdict were four times higher among juries who only saw the slow-motion version. Apart from the life or death decisions in the courtroom, the study also examined the impact of slow motion on the perceived intentionality of a “helmet-to helmet” incident in American football. Participants felt that the action was significantly more intentional if they saw it in slow motion compared to real time. Contrary to our focus here, this study relied on laypeople and not on sport experts or referees to assess the incidents. Moreover, a scale in the range of 0–100 without a clear link to decisional criteria of referees was used to measure perceived intentionality. Therefore, it is difficult to assess whether these findings generalize to expert decision-making in sports and refereeing. Indeed, literature on the influence of video speed on the decision-making process by referees remains sparse. This is remarkable given the importance of referee decisions and the increasing reliance on slow-motion video to evaluate these decisions.

Lorains, Ball, and MacMahon (2013a) showed that elite athletes in Australian football outperform sub-elite and novice groups in off-field decision-making when the stimuli were shown at increasing video speeds. Above real-time video speed would allow for faster processing, enabling experts to perform at a higher level of automaticity. In a follow-up study, Lorains, Ball, and MacMahon (2013b) used a training intervention to assess whether Australian football athletes would benefit from above real-time video speed to improve on an off-field decision-making task. The authors showed this was indeed the case, with the above real-time group outperforming a group that was trained on normal video speed and a group that received no training. In contrast to these studies, Gilis, Helsen, Catteeuw, Van Roie, and Wagemans (2009) observed that judgments of offside situations were significantly worse for assistant referees in association football when played at a faster compared to a slower speed. Put et al. (2016) extended these findings by applying a training intervention using different video speeds manipulations. They observed that only the group of assistant referees that was trained on decreasing video speeds showed an improvement in decisional accuracy for offside situations.

The previous studies focused mostly on decision-making by athletes or assistant referees in situations for which a more or less objective ground truth (pass or shoot; on- or offside) can be determined. To date, only a single study assessed whether slow-motion replays impact the decision-making process of association football referees in more ambiguous situations such as foul-play situations (Spitz, Put, Wagemans, Williams, & Helsen, 2017). These authors used custom-made video clips consisting of foul/no foul situations (corner kick and open play). They observed that slow motion yields more accurate technical decisions (foul versus no foul) – especially for corner kick situations, in which there are usually many possible incidents between pairs of defenders and attackers, but no difference was observed for disciplinary decisions (i.e. no card, yellow card, or red card). As expected, expert referees performed better compared to their less expert colleagues.

Building on this work, the current research aims to specifically address questions that previous studies left unanswered. First, the previous study by Spitz et al. (2017) used custom-made recordings of foul-play situations. Although such an approach provides better stimulus control regarding factors such as in-game perspective, balance of different types of foul-play situations, or team outfit, the stimulus material is necessarily limited to situations that are easy to re-enact, which might not necessarily generalize to the type and intensity of foul-play situations observed during real football matches. Second, in this study we are specifically interested in decision-making regarding the most ambiguous type of foul-play situations that occur during a soccer game: tackle incidents. Although the stimulus set of Spitz et al. included tackling, this was only a small subset of the potential infringements also including pushing or holding an opponent. Third, in the current study we are not only interested in the decisional accuracy of referees. That is, although Spitz et al. did not observe an influence of video speed on decisional accuracy of disciplinary decisions, video speed might have a distinct influence on the directionality of these decisions (i.e. despite similar accuracy, referees might penalize situations more severely in slow motion). Fourth, Spitz et al. were interested in the influence of expertise on decision-making performance. In this study, we solely focus on the performance of international elite referees (i.e. comparable to the highest level of expertise used in Spitz et al.).

Thus, the goal of this study was to assess the impact of video speed on the quality of decision-making by international elite association football referees for foul-play situations (tackle incidents) derived from real, international football matches. As in Spitz et al. (2017), we relied on a referee-specific decision-making task to examine the biasing potential of slow-motion video footage. Specifically, referees from European top competitions determined the disciplinary decision (no card, yellow card, or red card) for 60 foul-play situations replayed in either real time or slow motion. The quality of referees’ decisions was assessed in two respects, accuracy and directionality of the decisions.

In addition to the applied significance of the current study, this experimental task also taps into the process of the perception of causality (i.e. what is the cause-effect relationship between the tackle of player A and player B falling on the ground?). This line of work goes back to the Gestalt psychological tradition (Heider & Simmel, 1944; Michotte, 1954, 1963), and much of Michotte’s seminal work still stands today (for a review, see Wagemans, van Lier, & Scholl, 2006). One of Michotte’s compelling demonstrations of the perception of causality is the launching effect. Here, two objects are presented to observers and one of them starts moving towards the other. As soon as the first object is adjacent to the second object, the second one starts moving. When presented with this sequence, observers spontaneously reported that the first object “launched” the second object (i.e. was responsible for the second object’s motion). Particularly relevant for the current study, Michotte also documented that the perception of causality was strongly influenced by the speed at which individual objects moved (Michotte, 1954, pp. 103–104). When this speed was substantially lower compared to the classic demonstration, observers no longer reported any launching percept and claimed that both objects were moving independently. As such, we can expect that the perception of causality in these slow-motion replays will strongly diminish or may be even absent. Furthermore, it is well known that the perception of causality induces binding effects over space and time (i.e. objects are perceived to be closer together and the event is estimated to last shorter) (Buehner & Humphreys, 2009, 2010). More generally, conditions in which spatiotemporal predictability is violated are estimated to last longer compared to predictable events (Eagleman, 2008). In line with Caruso et al. (2016), we hypothesize that this will lead referees to attribute more premeditation and intentionality to the actions they are reviewing, because they perceive the player as having more time to act. That is, although the clips are replayed at an objectively lower speed, the subjective interpretation of the duration of the action is increased due to the violations of spatiotemporal predictability associated with the replay of the clip. As such, we expect increased judgment of harmful intentions and therefore higher sanction levels (red instead of yellow card) after slow-motion replays.

## Methods

### Participants

A total of 139 active international elite referees from five European countries were invited by email to participate in the experiment. Eighty-eight male referees (mean age = 37.8 years, SE = 0.88) participated in the experiment, yielding a response rate of 63%. All were top-class referees active in European professional football. They provided informed consent and the study was approved by the local University ethics committee (G-2015 04 218).

### Stimuli

**Fig. 1:**
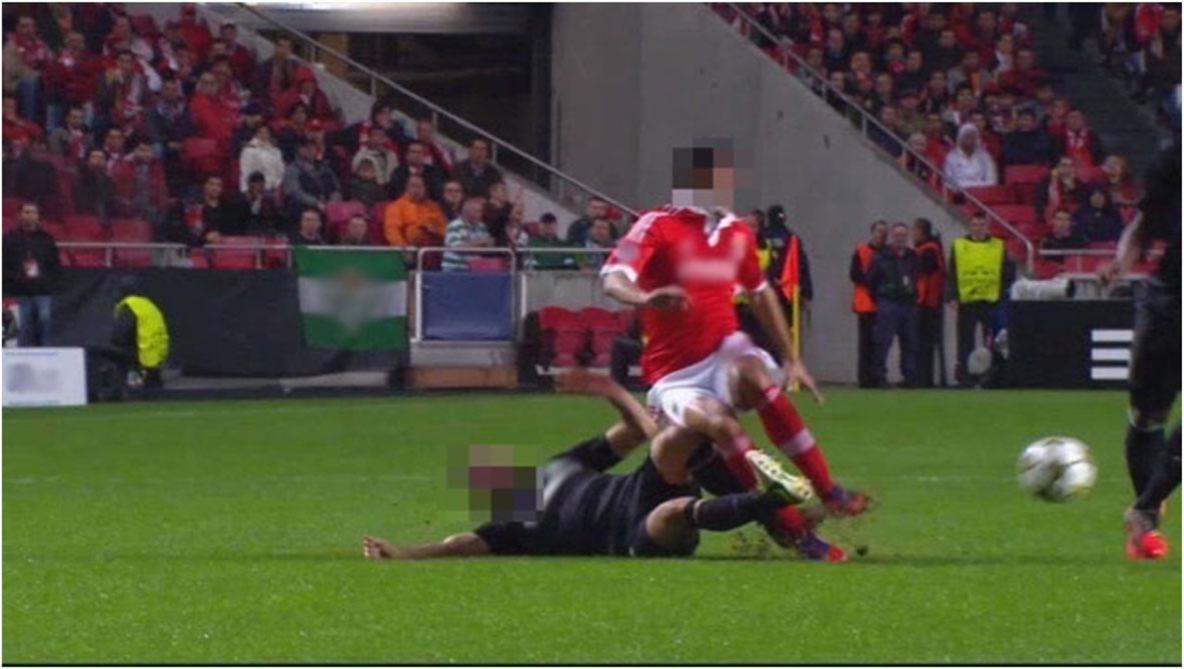
Example of a foul-play situation for which the referees had to make a disciplinary decision (no card, yellow card, or red card)

A total of 60 realistic and representative video clips of tackle incidents were selected. These foul-play situations were taken from matches of the UEFA (Union des Associations Européennes de Football). We took particular care in selecting specific video clips, in accordance with the following criteria:

(i) the foul-play situations are represented well in the video clip, as recorded from an in-game perspective (see Fig. 1 for a screenshot of an example);
(ii) the position of the players is clear, most of the time even in the middle of the field, to exclude the influence of the position or situation in the game;
(iii) the players are always clearly distinguishable.

Each video clip was edited by using two software programs (Adobe Premiere and Final Cut Pro). We manipulated the original video clips as follows: either we reduced the speed of a real-time video clip four times or we increased the speed of a slow-motion video clip four times. The video clips were cut down to the essential fragment to be able to come to a correct decision. This resulted in two identical video clips for each situation, 60 video clips in real time (mean duration 3.08 s) and the same 60 video clips in slow motion (mean duration 12.32 s). The same information was present in both video speed conditions, only the temporal dynamics were modulated. The video clips are MP4 files (720 × 406 pixels), with good quality and with the background sound removed. Two independent and experienced ex-international referees, still involved as referee match observers for UEFA, determined the reference decisions based on the rules established by Law 12: Foul and Misconduct (FIFA, 2016). Both modes (real time and slow motion) were available to determine the reference decisions. These two referees were able to view the clips multiple times and they knew the decisions that had been made by the original referees during the game. As an expert panel, both referees made independent evaluations first and then discussed the video clips with the UEFA chief refereeing officer to resolve any disagreement. They reached the following consensus decisions (reference decisions): 4 = no foul; 2 = foul with no card; 36 = foul with yellow card; and 18 = foul with red card.

### Procedure and design

The experimental video clips were presented to the participants via a web-based application (perception4perfection), developed by the research unit “perception and performance” of the KU Leuven to be used for perceptual-cognitive skills training and research. All participants received an account with an individual login and password to enable them to assess the different situations at their own place. Each referee assessed 60 unique situations. Half of the participants – randomly selected – evaluated 30 situations in real time and 30 situations in slow motion. The other half of the group evaluated the same situations but in the different video speed condition (real time instead of slow motion and vice versa). Three different video clips were used for familiarization with the test. During the test, every slow-motion video clip was followed by a real-time video clip (and vice versa) and after every 20 video clips there was a break. The participants watched each video clip just once, either in real time or slow motion. After every video clip, the referees had to assess whether a foul occurred (technical decision) and they had to indicate the disciplinary decision (no card, yellow card, or red card) within a time window of 10 s by clicking on the mouse. After clicking, no correction was possible and there was no feedback. The referees were asked to assess the 60 situations, which took approximately 20 min, all on the same day.

### Data analysis

For all analyses, we used the R statistical programming language (version 3.3.2) and RStudio (an IDE for R, version 1.0.136). For data processing, we relied on the *tidyverse* package (Wickham, 2017) and for data visualization we used *ggplot2* (Wickham, 2009). All cumulative link mixed models were fit using the package *ordinal* (Christensen, 2015).

Due to the low number of no foul technical decisions, we decided to analyze the disciplinary decisions only. We collapsed the no foul and foul/no card situations into a no card category. Thus, all analyses pertained to disciplinary decisions that could take on the value no card, yellow card, or red card. We evaluated the performance of the referees in two different ways (cf. two aims formulated earlier). First, we computed a simple accuracy measure (i.e. whether the decision was the same as the reference decision or not) to test whether video speed influenced the accuracy of the decisions. It should be noted that this measure of accuracy is merely a reflection of how strongly the referee and reference decisions overlap, rather than being a quantification of how good the referees are at determining a certain objective characteristic of the video clips. An accuracy score was calculated for each video speed and referee; a paired t-test was used to examine differences between both video speed conditions (α = 0.05).

We complemented the analyses on accuracy with an analysis that aims to model the data directly and can take into account other aspects of the data, such as the direction in which the decision of the referees shifted. This statistical method is known as cumulative link mixed modeling or mixed ordinal regression. A mixed model allows to simultaneously model different sources of random variability present in the dataset. Importantly, for our experiment, these random effects comprise referees and stimuli. We always included random intercepts for both referees and stimuli, as well as random slopes of video speed condition for both referees and stimuli, and random slopes for reference decision for referees. This was the maximal random effects structure that guaranteed stable convergence during model fitting (Barr, Levy, Scheepers, & Tily, 2013). As predictors, we included reference decision and video speed, and the interaction between these variables. It should be noted that the reference decisions act as a kind of “manipulation check” variable. That is, we expect that as the reference decision increases in severity, the probability of making a more severe disciplinary decision also increases. If we do not observe a main effect of reference decision in our final model, this would imply that the referee decisions could not be predicted at all by reference decisions. To arrive at a final model, we used a model selection approach where we started from the most complex model including reference decision, video speed, and their interaction as predictors and used drop-in-deviance tests to assess whether simpler models fit the data better compared to the most complex model. For example, a model including main effects of reference decision and video speed as well as random intercepts of referee and stimuli has the following form:

$$ {\displaystyle \begin{array}{l} logit\left(P\left({Y}_i\le j\right)\right)={\vartheta}_j-{\beta}_1\left( Reference\ {decision}_i\right)-{\beta}_2\left( Video\ {speed}_i\right)-u\left({Referee}_i\right)-v\left({Stimulus}_i\right)\\ {}\kern23.5em i=1,\dots, n,\kern2.50em j=1,\dots, J-1\end{array}} $$

We model the cumulative probability of the *i*th referee decision falling into the *j*th category (i.e. no card, yellow card, or red card). *ϑ*_*j*_ are known as cut-points or threshold parameters. The inverse logits of these threshold parameters indicate the baseline cumulative probabilities for a response ending up in a certain category (or lower) when all predictors are set to zero. In our case, this implies baseline cumulative probabilities for the clips belonging to the no card reference condition shown in real time (see the “Results” section for an example using the estimated threshold coefficients). The sign of the regression coefficients for reference decisions and video speed indicates how the cumulative probabilities are influenced. For example, positive coefficients indicate that the value of these predictors is associated with a higher category rating (i.e. a more severe decision).

## Results

### Accuracy

**Fig. 2:**
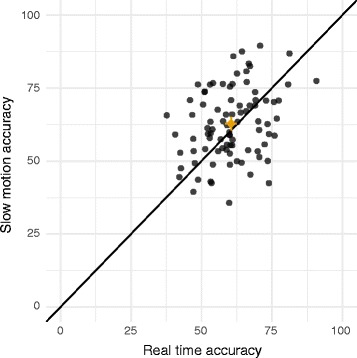
*Scatterplot* of the individual accuracy data in percentage. The accuracy for the slow-motion condition is plotted against the accuracy for the real-time condition. *Dots* indicate individual referees and the solid line indicates the identity line. The *orange dot* indicates the mean accuracy across referees. The *orange lines* indicate bootstrapped 95% confidence intervals for the mean accuracy in both conditions

As can be derived from the scatterplot in Fig. 2, the task proved quite difficult in both conditions (slow motion: M = 63%, SD = 12; real time: M = 61%, SD = 10). There was no significant difference between the accuracy scores in the slow-motion condition compared to the real-time condition (t(87) = − 1.60, *p* = 0.11).

### Cumulative link mixed modeling

As highlighted in the “Data analysis” section, we used a top-down model selection approach starting with the most complex model, and reducing it in complexity until the drop-in-deviance test indicated that there was no longer a benefit to simplifying a model. Table 1 summarizes the output of this model selection process. As can be derived from Table 1 (top row), a full model (main effects and interaction) was not preferred over a main effects only model. The main effects model, however, was preferred over the model including only video speed (bottom row) or only reference decision (middle row).

**Table 1:** Results of the model selection process

Comparison	LR statistic	df	*p* value
Full vs. main effects	2.79	2	0.25
Main effects vs. reference	15.72	1	< 0.0001
Main effects vs. video speed	41.98	2	< 0.0001

The test statistic (LR) is the likelihood ratio statistic (i.e. the ratio of the log-likelihoods of both models). This test statistic is (asymptotically) Chi-square distributed (with df mentioned in the column). The *p* values are thus derived from this distribution

**Fig. 3:**
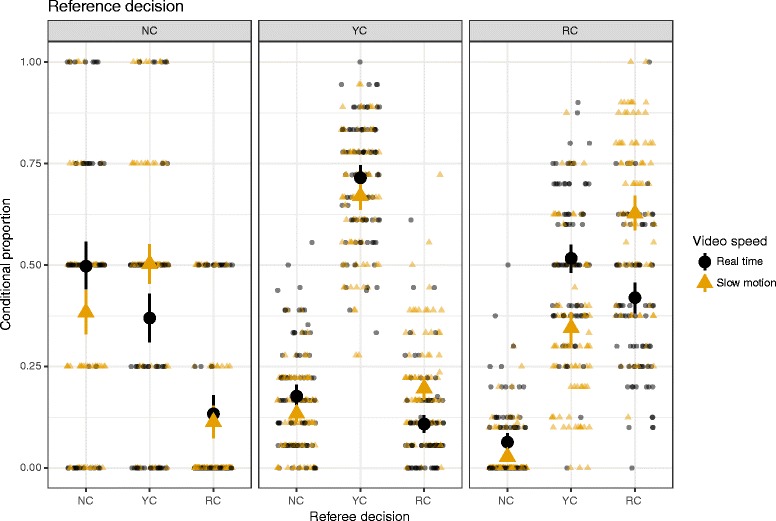
Conditional proportion of responses for each combination of reference decision and video speed condition. Each set of three *dots* indicating the combination of reference decision and video speed thus sums to 1. The error bars represent bootstrapped 95% confidence intervals. NC no card, YC yellow card, RC red card

Thus, Table 1 shows that the best model was one including both a main effect of the reference decision and video speed, yet no interaction between both factors. This implies that both the reference decision and video speed have an effect on the severity of the disciplinary decisions of the referees. The parameter estimates of the main effects model are summarized in Table 2, in terms of the model coefficients and threshold coefficients. The values of the threshold coefficients indicate the predicted cumulative probabilities (in logit units) for choosing a certain response category (or lower) for the “no card” reference decision shown in real time (i.e. the baseline condition). Taking the inverse logit of both estimates shows that the cumulative probability for choosing the “no card” category is 0.49 (i.e. exp.(− 0.03) / (1 + exp.(− 0.03))) and 0.99 for the “yellow card” (or lower) category. Positive estimates of the model coefficients can be interpreted as increased odds for choosing a higher category in the dependent variable (i.e. here, the referee decision). The estimates of the yellow and red card reference decisions thus imply that there are increased odds for choosing a higher category compared to the no card reference decisions. That is, reference decisions and referee decisions are tightly linked. Critically, the estimate is also positive for the slow-motion versus real-time condition. Thus, slow-motion clips are associated with increased odds for choosing a higher category on the decision scale. Figure 3 depicts these results graphically. Here, conditional proportions of responses are depicted in function of reference decision and video speed. For example, in the case of a real-time depiction of a yellow card reference decision, the proportion of yellow card responses is highest, followed by no card responses and red card responses. In contrast, in the slow-motion condition, this situation changes such that yellow card responses are slightly less numerous, no card responses also decrease, and red card responses increase. Similar patterns are observed for the no card and red card reference decisions.

**Table 2:** Parameter estimates for the main effects model

	Estimate	Standard error
Model coefficients		
Yellow card	1.84	0.60
Red card	4.29	0.64
Slow motion	0.90	0.21
Threshold coefficients		
NC|YC	− 0.03	0.57
YC|RC	4.66	0.57

NC no card, YC yellow card, RC red card

## Discussion

An increasing number of competitive matches in professional sports are recorded on video and referee decisions are retrospectively analyzed by coaches, fans, players, and sport commentators using slow-motion replays. In March 2018, the International Football Association Board (IFAB), the game’s law-making body, approved, with immediate effect, the use of video assistant referees in association football. A video assistant referee has to check every situation to examine whether a potential clear and obvious error has been made in a match-changing situation. The video assistant relies on video clips in slow motion and/or real time for that. The video assistant referee eventually informs the main referee who then has the opportunity to review footage on the field before making a final decision. It is often acknowledged that slow motion distorts reality and can change the way body movements and intentions are perceived (Caruso et al., 2016). In the current study, we examined the impact of slow-motion video clips on the assessment of foul-play situations.

We used 60 representative video clips of tackle incidents to assess decision-making performance. The decision-making task required an assessment of the intentionality and seriousness of the foul to determine the disciplinary sanction for the offending player. As highlighted in the “Results” section, the performance of the elite referees in this study was comparable with previously reported decisional accuracy scores for referees on the field of play (Gilis, Weston, Helsen, Junge, & Dvorak, 2006; Mascarenhas, Button, O’Hare, & Dicks, 2009) and based on video replays (Gilis et al., 2006; Spitz et al., 2017). The rather low accuracy scores in this study and previous publications are probably due to the difficulty of the situations and also to the fact that the Laws of the Game (FIFA, 2016) leave room for interpretation by the referee. In fact, the difference between the concept of a careless or reckless offence or an offence with excessive force that should be considered to whistle a foul and give a yellow or red card, respectively, still remains quite vague.

Our results demonstrate that decisional accuracy of referees was not significantly different in slow motion compared to real time. An explanation for the fact that slowing down the video speed did not add to decisional accuracy might be that it reduces the fidelity and representativeness of the everyday performance context (Hettinger & Haas, 2003; Stoffregen, Pagulayan, Smart, & Bardy, 2003). Lorains et al. (2013a) put forward the same argument to explain why elite athletes made more accurate decisions under faster speed conditions: faster speeds more closely replicate the cognitive processing demands required by sport athletes who need to make crucial decisions in dynamic and time-constrained environments. The same can be said for referees and expert performers in other domains (e.g. military, police, aviation). On the other hand, for referees, it can be argued that the impact of slow motion on decisional accuracy depends on the type of decision and the type of situations that need to be assessed. For more objective assessments, such as offside decisions in case of assistant referees, slow motion might be of added value and increase decisional accuracy. Indeed, contrary to the findings with elite athletes, Gilis et al. (2009) observed that offside decisions were significantly more accurate when played at a slower compared to a faster speed. These offside decisions require a more objective assessment of spatial (i.e. how are the players positioned relative to one another) and temporal (i.e. the exact moment of the pass) landmarks. Furthermore, Spitz et al. (2017) showed that elite referees were more accurate in slow motion for technical decisions and in case of foul-play situations with multiple players involved and several potential foul plays at the same time. In these situations, slow motion might make it easier to select the relevant information, to see whether there is actual contact, and to identify the offender and exact location of the foul. However, the results of Spitz et al. also showed that slow motion does not add to decisional accuracy for typically more ambiguous tackle incidents and the determination of the disciplinary sanction for the offending player. These results were replicated in the current study and it seems that the impact of slow motion on decisional accuracy depends on the type of decision and situation.

Although highly valued, an analysis of accuracy scores (% correct decisions) may be deceptive because it is not always a good indicator of what people are doing and does not provide a complete and comprehensive account of performance differences. The decisions that yield a given accuracy level may encompass extremes of liberal and conservative biases. To gain more insight into the underlying mechanisms of slow motion on the perception of foul play and to determine how the decisions deviated from the reference decision, we performed a mixed ordinal regression analysis. As such, we were able to determine whether the speed of the video replay biased the decisions in a certain direction.

The results indicate that slow-motion video clips are associated with increased odds for choosing a higher category on the decision scale (i.e. no card, yellow card, or red card). In case of high-impact tackle incidents, there is a clear impact of slow motion, altering the judgment of the referees towards more severe disciplinary sanctions for the offending players. These results are in line with previous research investigating the way replay speed affects human judgment in the courtroom. Viewing a situation in slow motion, compared with regular speed, increased the perceived intent of a violent action (Caruso et al., 2016). A main characteristic of slow motion is that it affects the impressions of the duration over which real-time events unfold. As suggested by Caruso et al., the temporal modulation of the dynamics creates the perception that the offender has much more time to contemplate his action than he actually does. Therefore, physical contacts and violent actions might be perceived more intentionally and seriously. Indeed, we hypothesized that slow-motion replays could disrupt normal perception of causality (Michotte, 1954, 1963), which in turn could influence the perceived duration of the event (on top of the fact that it already was replayed in slow motion). In line with the reasoning outlined in Caruso et al. (2016), this would create a situation in which observers attribute more premeditation to the player’s action. More generally, this view is consistent with a framework in which humans continuously generate predictions based on the incoming sensory information, where predictions are tuned to physics and biomechanics of our world and its associated time constants (Richmond & Zacks, 2017). In the case of slow motion, these predictions are violated, which in turn might influence the subjective duration of the event. Indeed, spatiotemporal predictability has been shown to influence subjective duration such that unpredictable events are perceived to last longer. Thus, in this case, as slow motion alters the fit of the stimulus to those time constants, this could lead viewers to associate the player’s action with a higher degree of intentionality.

It should be noted that our speed manipulation might also change more central aspects such as density of information or visual saliency. The temporal manipulation of video speed might shift attention to different relevant aspects of the visual display parts because the manipulation raises perceptual saliency of different aspects (Fischer, Lowe, & Schwan, 2008). The effect we reported could thus be the result of differential focus on relevant aspects of the visual display between both speed conditions. Indeed, eye movement studies have shown that looking behavior in dynamic scene viewing shows two distinct phases. That is, an ambient style for initial explorative looking and a focal style for subsequent, more detailed scrutiny (Eisenberg & Zacks, 2016). Thus, follow-up studies are needed to further investigate the underlying mechanisms of the slow-motion effect. Not only eye-movement registration, but also verbal reports, spatial occlusion paradigms, and manipulations to equate presentation duration for both speed conditions are viable methodologies to disentangle and explain the effect of video speed on referees’ decisions. Moreover, future studies could manipulate presentation speed of the different segments within a clip (before and after the foul) independently to know whether slow motion affects the cause (pre-contact) and effect (post-contact) of the foul in a similar way.

Our findings on the biasing potential of slow-motion replays are relevant in light of current evolutions and innovations within team sports in general and association football in particular. Fans, sport commentators, the media, and (video assistant) referees more and more make use of technology and they only see videos and replays in slow motion. Despite the fact that referees on the field of play have to decide in real time, these slow-motion replays are adopted as objective representations of the foul-play situations. However, our results demonstrate that it is important to take the biasing potential that results from the artificial distortion of temporal dynamics into account when assessing foul play situations. Slow motion can make the offence look more pre-meditated than it actually was and can, for example, change the disciplinary sanction from a yellow card into a red card.

Slow motion can therefore not be seen as a valid basis of comparison and we recommend to only use real-time footage for judging the amount of risk for the opponent’s safety involved and the perceived impact/intent of a tackle. Reminding people that they are watching a slow-motion situation might not be sufficient since previous research has shown that even when people are aware that there is an incidental influencing factor (e.g. slow motion), they often do not correct sufficiently (Epley, Keysar, Van Boven, & Gilovich, 2004; Gilbert, 1989).

In this study, an expert panel of ex-referees determined the reference decisions for the situations. Both modes (real time and slow motion) were available to determine the reference decisions and we were not able to verify whether the expert panel based their reference decisions more on the slow motion rather than on the real-time presentation mode. A potential alternative interpretation of our results could thus be that the influence of slow motion on referee decisions reflects that they converge towards the expert panel decisions. We see two arguments that counter this alternative interpretation. First, it has been shown that showing an action at both regular and slow-motion speed is effective in reducing the possible biasing influence of slow motion (Caruso et al., 2016). Second, such an account would predict that the referee decisions would converge towards the reference decisions in the slow-motion condition which was arguably not the case (as depicted in Fig. 2). Future research could focus on the extent to which video speed influences the determination of the reference decisions by the expert panel. Furthermore, it would be interesting to examine the impact of several other potentially modulating variables, such as the number, the order, the duration, and the viewing angle of replays on the assessment of foul-play situations. In line with the findings of Caruso et al. (2016), we predict that the difference between slow and regular speed remains over multiple viewings of the same foul incident. On the other hand, the difference between slow motion and real time could decrease over multiple viewings of the same foul play (as referees might pick up on information after multiple viewings at regular speed that they had originally missed), but the bias could instead also get more pronounced the more often referees viewed the foul. A better insight in these aspects is definitely of interest for many people involved in modern football.

In the context of football refereeing, the distinction between perception and judgment has important implications for the nature of the errors they are making and for how to learn to correct them. For instance, with respect to offside judgment, the perceptual nature of the flag errors (unnecessarily signaling offside because the most advanced offender is perceived as being ahead of his actual position) has given rise to training programs where assistant referees are taught to cognitively compensate for their perceptual mistakes (e.g. Catteeuw, Gilis, Wagemans, & Helsen, 2010; Put, Wagemans, Spitz, Williams, & Helsen, 2015). A similar training program appears to be needed for those who watch fouls in slow motion, when it comes to making a judgment of the severity of the tackle and the required sanction (yellow versus red card).

## Conclusions

In this study, we explored whether it is justifiable to analyze and compare the in-game decision of the referee with decisions based on a complete different viewing mode, i.e. at slow-motion video speed. For certain types of situations and decisions, slow-motion video can be a helpful tool and be of value to increase decisional accuracy. By slowing down an image, it might become clear who initiated a foul, whether there actually was contact and whether a foul occurred either inside or outside the penalty area. However, judging human behavior and human emotion, such as intentionality, is quite another story. Based on our results we conclude that slow motion has an impact and can make the difference between perceiving an action as careless (no card), reckless (yellow card), or with excessive force (red card). Therefore, caution is warranted before adopting video technology and clear guidelines should be defined (Collins, 2010; Royce, 2012). Our findings have significant implications for the current debate over the introduction of technology and for setting guidelines regarding the use of slow motion in the decision-making process.
